# Editorial: Immigration in the Global Era: Migrants and the People and Laws at Origin and Destination

**DOI:** 10.3389/fsoc.2021.740324

**Published:** 2021-07-28

**Authors:** Guillermina Jasso, Moshe Semyonov

**Affiliations:** ^1^ Department of Sociology, New York University, New York, NY, United States; ^2^ Department of Sociology and Anthropology, Tel Aviv University, Tel Aviv, Israel

**Keywords:** migrant selectivity, integration of immigrants and their descendants, impacts of immigration, laws on exit and entry, public attitudes toward migrants in both origin and destination countries

The world of migration is a vast and diverse landscape. The 17 articles in this collection cover every step in the migration process, from origin to destination and beyond, as migrants in a variety of streams – temporary and permanent; labor, family, and refugee; of diverse countries, backgrounds, and aspirations – find help and hindrance, welcome and opposition from a wide swath of nonmigrants and immigration officials. Scholarly topics include self-selection and government-selection, into legal status and residential location; determinants of prejudice; integration and naturalization; children and subsequent descendants of migrants; impacts of law and contexts of reception. The articles traverse theory and empirics, micro and macro matters, qualitative and quantitative approaches, cross-sectional and longitudinal perspectives, single-country and multi-country settings.

## Selectivity and Law, From First Steps to Naturalization

Soysal and Cebolla-Boado address selectivity in four usually unobserved but here self-described traits – ambition, creativity, independent-mindedness, and risk-taking – among international higher-education students from China in Germany and the United Kingdom, comparing them to nonmigrant higher-education students in China, Germany, and the United Kingdom. Results indicate no systematic differences across these student groups and no gender difference, suggesting global convergence in these traits and a remarkable homogeneity around the world in how university students see themselves.

Jacobs examines how skilled migrants navigate legal migration channels in the United States, based on a sample from India holding H-1B temporary work visas, plus smaller subsamples of unsuccessful H-1B applicants and immigration lawyers. The sample includes both direct recruits newly arriving in the United States and H-1B visaholders transitioning in the United States from a student (F-1) or exchange (J-1) visa. Results document the challenges and uncertainties of the visa process, noting how an earlier transition (e.g., from F-1 to H-1B) increases the likelihood of applying for legal permanent residence (LPR), now with more options beyond employment visas (e.g., via marriage), but also how the mismatch between LPR aspirations and the temporary H-1B visa may lead to emigration.

Spörlein and Kristen assess educational selectivity among labor migrants from 15 countries in Africa, Latin America, Asia, and Europe who moved to Germany, England, Ireland, the Netherlands, and Spain, together with refugees from Afghanistan, Iraq, and Syria in Germany. Results indicate that there are few selectivity differences between labor and refugee migrants, but these differences vary, with some labor migrant groups scoring the same or lower on selectivity than refugees; moreover, every origin group includes both positively and negatively selected individuals.

Haberfeld et al. assess post-migration selectivity in location choices among refugees who arrived in Sweden from nine countries in Africa, Asia, and Europe during 1990–1993, a period when refugees were assigned an initial location but were subsequently free to move. Analysis of register data over an eight-year period indicates that refugees’ educational levels are related to their destination choices, with highly skilled refugees more likely than the less skilled to move to labor markets with more opportunities, and that all relocation choices have positive effects on refugees’ income growth.

Jasso develops a framework for analyzing migration restriction regimes, applicable to any migration stream and any country, illustrating it with the United States system for LPR. Restriction may be based on personal criteria and/or numerical ceilings and generates both backlogs and unauthorized migration. The framework can be used to summarize a country’s migration law -- rules for admission, periodization of its immigration history, and policy devices for addressing backlogs and unauthorized migration -- as well as to compile stocks and flows of legal temporaries, LPRs, and naturalized.

McAvay and Waldinger analyze naturalization patterns among migrants in France from over 50 countries who are married to a French citizen and thus, under French law, eligible for both standard naturalization and a fast-track naturalization for spouses of French citizens, which has more extensive documentary requirements, especially with respect to demonstrating that the marriage is genuine. Results indicate that while marriage to a French citizen is the single most powerful determinant of naturalization, choice of track is strongly related to the nativity and parentage of the French citizen spouse, with marriage to a French native especially likely to promote marriage-track naturalization, particularly among women.

## Prejudice and the Contexts of Reception

Bohman et al. explore the effect of political discussions with peers during adolescence on prejudice against immigrants in Sweden, using data on two cohorts followed for a five-year period (ages 13 and 16 at the start of the survey). Results show an association between political discussion and prejudice, its strength increasing as the adolescents grow older. Moreover, the effect of political discussions depends on the level of prejudice among peers; political discussion with low-prejudice peers is associated with lower levels of prejudice, but political discussion with high-prejudice peers is not significantly related to attitudes toward immigrants.

Mitchell examines how differences in social trust, both within and between countries, influence attitudes about immigrants, using the European Social Surveys of 2002–2016 and, for robustness checks, supplementary data from the European Values and World Values Surveys. Results from longitudinal analyses indicate that countries with higher levels of social trust have more favorable attitudes toward immigrants and that changes in trust over time, albeit small, result in comparably large changes in anti-immigrant attitudes, even when controlling for other social factors.

Gorodzeisky and Semyonov analyze opposition to immigration among natives of 20 countries in the 2014 European Social Survey, showing that opposition varies not only across host countries but also across five ethnoreligious migrant groups (of the same or different race/ethnicity as a majority population, plus Jews, Muslims, and Roma). Results indicate hierarchical opposition to the five groups, being most extreme toward Muslims and Roma but relatively minor toward immigrants of the same race-ethnicity or toward Jews, as well as varying sources of opposition to immigration (e.g., threat of competition, fear of crime, racism, intergroup contact).

Evans and Kelley analyze prejudice toward outgroups – using a classic measure of social distance rooted in the work of Bogardus that taps objection to members of specific groups “as neighbors” -- among respondents in 100 countries (using the World Values Surveys, European Values Surveys, and European Quality of Life Surveys). They find that prejudice and social distance against immigrants, other races, Hindus, Jews, Muslims, and Roma tend to decline with level of socioeconomic development of the host country. They also find that a single latent ethnoreligious prejudice generates prejudice against specific outgroups.

Steele and Perkins examine how natives’ perceptions and misperceptions about the size of the noncitizen population affect attitudes toward redistribution and social policies among a sample of residents of New York City – one of the most diverse and ethnically heterogeneous cities in the world -- recruited via Amazon Mechanical Turk. Results indicate that about a quarter of New Yorkers overestimated the relative size of the noncitizen population in their neighborhood, and these respondents were among the least supportive of redistribution and other social policies.

## Life in the Destination Country

Bevelander and Luik assess the employment integration of refugees in Sweden in the first 12 years since arrival, using longitudinal data on three arrival cohorts (1998–2000). Despite differences across the three main refugee groups (from three countries in East Africa, one from Europe, and four from the Middle East), results show similar patterns of employment integration; all refugee groups increase their employment probabilities, but from different starting points and at different speeds, some reaching parity during the study period. In particular, women from Bosnia, Eritrea, and Ethiopia have similar or higher employment probabilities than Sweden-born women after between five and eight years in the country.

Amit and Chachashvili-Bolotin analyze the mismatch between subjective work perception and actual position in the labor market – the discrepancy between actual integration (combining actual job and wage) and subjective integration (combining satisfaction from job and wage) – in Israel among four immigrant groups (from Ethiopia, the Former Soviet Union, and Western countries, plus pre-1989 immigrants) and four native-born groups (second-generation Ashkenazim and Mizrahim, third-generation, and Arabs). A larger fraction of women than of men experienced a positive mismatch, and a smaller fraction of women than of men experienced a negative mismatch; moreover, the largest fractions experiencing a positive mismatch occurred in groups with disadvantaged backgrounds.

Kogan and Shen assess life satisfaction among migrants from/to 30 European countries, using data from the 2008–2016 European Social Surveys. Results indicate that immigrants’ assessments of economy, democracy, and quality of public goods (such as health and education systems) in the host country contribute to life satisfaction, with satisfaction with the economy being the strongest correlate of life satisfaction, particularly among migrants from Turkey and countries of Eastern and Southern Europe.

Wilkes and Wu study the complex relations between three kinds of perceived discrimination (based on race, ethnicity, or anything including race/ethnicity) and three types of trust (generalized, specific, political) between five groups in Canada (Canada-born whites, Canada-born people of color, Indigenous people, foreign-born whites, foreign-born people of color). They find that perceived discrimination is more relevant to general and specific trust than to political trust and that perceived discrimination explains more of the trust gap between foreign-born people of color and the native born than between foreign-born whites and the native born.

Lubbers and Gijsberts use a four-wave panel to assess change in self-rated health among immigrants from Bulgaria, Poland, Spain, and Turkey who arrived in the Netherlands in 2012 and 2013. Results indicate that lack of Dutch friends, perceived discrimination, cultural distance, and homesickness strongly affect self-rated health; moreover, self-rated health declined over time, although slightly, mostly from “very good” to “good,” and possibly linked to the new immigrants becoming parents in the early years after migration.

Sanderson and Kentor assess the relation between migration and development by analyzing migration (im)balances and wage differentials within 22 pairs of countries in the Americas at five decennial points between 1970 and 2010. They find a positive feedback between international migration and cross-national inequalities; wage differentials between countries have a strong effect on migration, especially in contiguous countries, while migration has a smaller effect on wage differentials.

## Going Forward

The 17 articles open new doors for further inquiry, as migration continues and researchers study its impacts on the well-being of migrants and others at origin and destination.

